# Comment on “Web-Based Measure of Life Events Using Computerized Life Events and Assessment Record (CLEAR): Preliminary Cross-Sectional Study of Reliability, Validity, and Association With Depression”: Validity and Methodological Issues

**DOI:** 10.2196/14505

**Published:** 2020-05-21

**Authors:** Jamal Rahmani, Roya Karimi, Farideh Mohtasham, Siamak Sabour

**Affiliations:** 1 National Nutrition and Food Technology Research Institute Shahid Beheshti University of Medical Sciences Tehran Iran; 2 Department of Clinical Epidemiology School of Health and Safety Shahid Beheshti University of Medical Sciences Tehran Iran; 3 Department of Clinical Epidemiology School of Health and Research Institute for Gastroenterology and Liver Diseases Shahid Beheshti University of Medical Sciences Tehran Iran; 4 Safety Promotions and Injury Prevention Research Centre Shahid Beheshti University of Medical Sciences Tehran Iran

**Keywords:** validity, methodological issues, diagnostic test

We were interested in the article titled, “Web-Based Measure of Life Events Using Computerized Life Events and Assessment Record (CLEAR): Preliminary Cross-Sectional Study of Reliability, Validity, and Association With Depression” published in *JMIR Mental Health* [1].

One of the aims of the abovementioned study was to assess the validity of Computerized Life Events and Assessment Record (CLEAR), considering the Life Events and Difficulties Schedule (LEDS) and the List of Threatening Experiences Questionnaire (LTE-Q) as gold standards among 328 participants (126 students; 202 matched midlife sample: 127 unaffected controls, 75 recurrent depression cases). The authors concluded that CLEAR has acceptable validity and great potential for effective use in research and clinical practice. However, there are some methodological issues in this conclusion that are mentioned below.

First, there are some measures that can be applied to the assessment of the validity of a test including sensitivity, specificity, positive predictive value, negative predictive value, positive likelihood ratio (LR+; ranging from 1 to infinity; the higher the LR+, the more accurate is the test), negative likelihood ratio (LR–; ranging from 0 to 1; the lower the LR–, the more accurate is the test), and odds ratio (ratio of true to false results) [2-5]. According to the results, sensitivity of CLEAR was 59.1% and 43.1% compared to LEDS and LTE-Q, respectively, as gold standards. Likewise, specificity of CLEAR was 65.4% and 78.6%, respectively, compared to the abovementioned gold standards.

It is good to know that sensitivity is an important measure in public health aspects instead of clinical fields. Likewise, the positive predictive value and negative predictive value are among measures that are more appropriate for advice about the validity of a diagnostic test for clinical purposes [3-5]. Therefore, we suggest applying predictive values, likelihood ratios, odds ratio, and diagnostic accuracy to decide the validity of CLEAR. Moreover, according to the data of study, LR+, LR–, odds ratio, and diagnostic accuracy of CLEAR will be 1.6, 0.6, 2.6, and 62%, respectively, compared to LEDS (Tables 1 and 2) and 1.9, 0.7, 2.6, and 60%, respectively compared to LTE-Q (Tables 3 and 4). Therefore, there is a high level of uncertainty for decisions based on these values, and there is insufficient evidence to conclude that the validity of the CLEAR test is acceptable.

**Table 1:** Two by two table of Computerized Life Events and Assessment Record compared to Life Events and Difficulties Schedule as the gold standard.

CLEAR^a^	LEDS^b^ (gold standard)		
	Positive	Negative	Total
Positive	59	35	94
Negative	41	65	106
Total	100	100	200

^a^CLEAR: Computerized Life Events and Assessment Record.

^b^LEDS: Life Events and Difficulties Schedule.

**Table 2:** Assessing the validity of Computerized Life Events and Assessment Record compared to Life Events and Difficulties Schedule as the gold standard.

Parameter	Estimate
Sensitivity (%)	59
Specificity (%)	65
Positive predictive value (%)	63
Negative predictive value (%)	61
Diagnostic accuracy (%)	62
Likelihood ratio of a positive test	1.6
Likelihood ratio of a negative test	0.6
Diagnostic odds	2.6

**Table 3:** Two by two table of Computerized Life Events and Assessment Record compared to List of Threatening Experiences Questionnaire as the gold standard.

CLEAR^a^	LTE-Q^b^ (gold standard)		
	Positive	Negative	Total
Positive	43	22	65
Negative	57	78	135
Total	100	100	200

^a^CLEAR: Computerized Life Events and Assessment Record.

^b^LTE-Q: List of Threatening Experiences Questionnaire.

**Table 4:** Assessing the validity of Computerized Life Events and Assessment Record compared to List of Threatening Experiences Questionnaire as the gold standard.

Parameter	Estimate
Sensitivity (%)	43
Specificity (%)	78
Positive predictive value (%)	66
Negative predictive value (%)	58
Diagnostic accuracy (%)	60
Likelihood ratio of a positive test	1.9
Likelihood ratio of a negative test	0.7
Diagnostic odds	2.6

## Abbreviations

CLEAR: Computerized Life Events and Assessment Record
LEDS: Life Events and Difficulties Schedule
LR–: negative likelihood ratio
LR+: positive likelihood ratio
LTE-Q: List of Threatening Experiences Questionnaire
